# A cluster-randomized controlled trial of a computerized antithrombotic risk assessment tool to optimize stroke prevention in general practice: a study protocol

**DOI:** 10.1186/1472-6963-14-55

**Published:** 2014-02-07

**Authors:** Beata Bajorek, Parker Magin, Sarah Hilmer, Ines Krass

**Affiliations:** 1 School of Pharmacy – Graduate School of Health, University of Technology Sydney, PO Box 123 Broadway, Sydney, New South Wales 2007, Australia; 2 Department of Pharmacy, Royal North Shore Hospital, St Leonards, New South Wales 2065, Australia; 3 Primary Health Care Research, Evaluation & Development (PHCRED), Discipline of General Practice, Newbolds Building, University of Newcastle, University Drive, Callaghan, New South Wales 2308, Australia; 4 Department of Clinical Pharmacology, Level 12, Kolling Building, Royal North Shore Hospital, St Leonards, New South Wales 2065, Australia; 5 Faculty of Pharmacy, University of Sydney, Building A15 Science Road, Sydney, New South Wales 2006, Australia

**Keywords:** Stroke, Atrial fibrillation, Warfarin, Bleeding, Antithrombotic therapy, General practice, GP, Decision support, Risk assessment

## Abstract

**Background:**

Therapy for stroke prevention in older persons with atrial fibrillation (AF) is underutilized despite evidence to support its effectiveness. To prevent stroke in this high-risk population, antithrombotic treatment is necessary. Given the challenges and inherent risks of antithrombotic therapy, decision-making is particularly complex for clinicians, necessitating comprehensive risk:benefit assessments. Targeted interventions are urgently needed to support clinicians in this context; the Computerized Antithrombotic Risk Assessment Tool (CARAT) offers a unique approach to this clinical problem.

**Methods/design:**

This study (a prospective, cluster-randomized controlled clinical trial) will be conducted across selected regions in the state of New South Wales, Australia. Fifty GPs will be randomized to either the ‘intervention’ or ‘control’ arm, with each GP recruiting 10 patients (aged ≥65 with AF); target sample size is 500 patients. GPs in the intervention arm will use CARAT during routine patient consultations to: assess risk factors for stroke, bleeding and medication misadventure; quantify the risk/benefit ratio of antithrombotic treatment, identify the recommended therapy, and decide on the treatment course, for an individual patient. CARAT will be applied by the GP at baseline and repeated at 12 months to identify any changes to treatment requirements. At baseline, the participant (patients and GPs) characteristics will be recorded, as well as relevant practice and clinical parameters. Patient follow up will occur at 1, 6, and 12 months via telephone interview to identify changes to therapy, medication side effects, or clinical events.

**Discussion:**

This project tests the utility of a novel decision support tool (CARAT) in improving the use of preventative therapy to reduce the significant burden of stroke. Importantly, it targets the interface of patient care (general practice), addresses the at-risk population, evaluates clinical outcomes, and offers a tool that may be sustainable via integration into prescribing software and primary care services. GP support and guidance in identifying at risk patients for the appropriate selection of therapy is widely acknowledged. This trial will evaluate the impact of CARAT on the prescription of antithrombotic therapy, its longer-term impact on clinical outcomes including stroke and bleeding, and clinicians perceived utility of CARAT in practice.

**Trial registration:**

Australian New Zealand Clinical Trials Registry: ACTRN12613000060741.

## Background

### Age, atrial fibrillation, and stroke

Stroke accounts for significant morbidity and mortality globally every year, including in Australia (where this study is being conducted) [1]. Advancing age is a key non-modifiable risk factor for stroke, and this is important to recognize given that 80% of stroke sufferers are over 60 years old and that 80% of stroke-related deaths occur in those over 75 years old [1]. As the population ages the incidence of stroke rises, doubling the disability burden by the year 2031 [2].

Atrial Fibrillation (AF), commonly dubbed the ‘a*rrhythmia of the millennium*’, is the commonest irregular heart rhythm encountered in clinical practice, and is reportedly most prevalent in the elderly population [3,4]. It is infamous for its propensity to form stroke-causing thrombi in the heart chambers (atria) that can embolize to other parts of the body; these thrombo-emboli can subsequently occlude the blood vessels and disrupt the circulatory supply through to the brain, causing ischemia, i.e., stroke [5].

Patients with AF have at least a 5 to 6-fold increased risk of stroke [6], and in the elderly the proportion of stroke attributable to AF is at least one-third [7-9]. Independently, ‘old’ age and chronic AF are major risk factors for stroke but, unfortunately, both are irreversible. This complex of factors significantly compounds the risk of stroke in older people with AF, and for this reason, stroke prevention in this population is now recognized as a global health priority.

### Stroke prevention therapy

To date, numerous large clinical trials and meta-analyses have provided convincing evidence that antithrombotics (anti-clotting agents) can prevent stroke in patients with AF [10,11]. Historically, antithrombotic therapy has been reliant on two key agents: warfarin (anticoagulant) and aspirin (antiplatelet). Warfarin has been reported to reduce the risk of stroke by approximately two-thirds, whilst aspirin is less effective, reducing the risk by about 20% [10].

### Underutilization of preventative therapies

Although highly effective antithrombotic medicines, specifically warfarin, are significantly underutilized in practice [12-16]; data from local Australian practice shows that this is especially true in the target at-risk older population, even in the absence of apparent contraindications [12]. A fear of side effects underpins this [17,18] because antithrombotics inherently increase the potential for bleeding. Therapy, therefore, requires careful patient selection, close patient monitoring (via blood tests), and regular dosage adjustment. This is particularly true for warfarin, as pharmacological characteristics render its use more challenging in patients with multiple medical comorbidities and/or polypharmacy (e.g., elderly) [19].

Unfortunately, the inconvenience of therapy has led some clinicians to cite “old age” *per se* as a contraindication to treatment without further risk/benefit assessment, leading to the underutilization of warfarin. In older people the risk of stroke is high but so too may be the risk of medication misadventure [20], giving clinicians a platform to question whether initiating life-saving anti-coagulant therapy is done at the expense of causing potentially life-threatening side-effects.

In more recent times, clinicians have been awaiting the availability of alternative agents (so-called ‘novel’ oral anticoagulants - NOACs) to overcome some of these known challenges of warfarin therapy. Whilst the newer agents present viable, effective treatment alternatives (e.g., dabigatran, rivaroxaban, apixaban), they are not without their own inherent risks [21]. Furthermore, the cost-effectiveness of these agents must be carefully considered, before widespread use in the at-risk population can be supported. For this reason, decision-making regarding stroke prevention in AF is still largely focused on the selection of warfarin (anticoagulant) versus aspirin (antiplatelet) therapy, and has been highlighted again in the recent Australian Government Review into Anticoagulation Therapies for Atrial Fibrillation [22]. In any case, the expanding armamentarium of treatment options for stroke prevention in AF has added to the complexity of decision-making in this clinical context.

### Optimizing stroke prevention in general practice

Primary care is the key to optimal stroke prevention, and past guidelines have stated that “*General Practitioners … are the key to better stroke prevention. What is needed is proactive opportunistic screening and risk management, and prompt action for two groups of patients: those with stroke/TIA symptoms and those with atrial fibrillation*” (National Health and Medical Research Council, NHMRC; (23)).

Although it is widely recognized that general practitioners (GPs) are well placed to facilitate stroke prevention [23], the EXAMINE-AF study conceded that management by a GP, compared to a cardiologist, continues to be a significant predictor for the underutilization of warfarin, even in high-risk patients with a prior history of stroke [24]. A national survey of Australian GPs reinforces that there is a need to support GPs in quantifying stroke risk versus bleeding risk, to eliminate misperceptions about risk and to reduce anxiety about “*acts of commission*” [18]. Careful patient selection via risk/benefit assessment is integral to decision-making here, but guidelines *per se* are ineffective as they fail to demonstrate how evidence can be actioned in individual patients [13,25].

In a previous study, it has been shown that an applied comprehensive risk assessment process can significantly improve the utilization of therapy [26]. Using evidence-based algorithms to facilitate the systematic review of individual stroke risk and bleeding risk (taking into account medical, functional, cognitive, medication-related, and social factors), the use of antithrombotics increased (59.6% versus 81.2%, p < 0.001). Overall, 36% of patients required changes to their existing therapy, with 76.9% of these being ‘upgrades’ to more effective treatment. Following the success of this intervention, the founding risk assessment algorithm has been integrated into a key Clinical Indicator (Indicator 1.6) in the NSW Therapeutic Advisory Group’s (TAG) “*Indicators for Quality Use of Medicines in Australian Hospitals”*[27].

Although highly effective, a limitation of the above approach is the reliance on a paper-based process that cannot be linked into an increasingly electronic and online health system. In response to clinician feedback, the CARAT, an electronic (web-based) Computerized Antithrombotic Risk Assessment Tool, has been developed to aid clinicians in selecting appropriate antithrombotic therapy in older persons with AF. Unlike paper-based guidelines, it facilitates a systematic review of risk factors and calculates the estimated risk versus benefit of therapy in an individual patient. The CARAT inputs are modeled on those used in a previous study [26] and current evidence [28].

An exploratory study has yielded feedback from clinicians highlighting the potential utility of CARAT in practice [29]. Overall the majority of clinicians: are satisfied with CARAT’s format (94%); agree with its recommendations when applied to patient cases (72%); and agree with its estimate of stroke and bleeding risk (>66%). Most (63%) clinicians (geriatricians, cardiologists, neurologists, hematologists) positively indicated that CARAT was at least “*somewhat useful*” for their clinical practice with 22% indicating it was ‘very useful’ because:

“Highlights functional elements that need to be considered. Multifactorial review is essential”

“Warfarin is not a lifelong decision; people can fail a trial of anticoagulation but embolic stroke is irreversible [this tool helps re-focus away from bleeding risk, highlights stroke risk]”

“May be useful for a less experienced doctor”

“This tool should ideally be applied in ED and result should go to local medical officer”

“Rapid calculation of risks is very useful”

“Bleeding risk assessment section is very useful”

Whilst these findings are encouraging, the utility of the tool is expected to be far greater in general practice given its focus on chronic disease prevention and management, and where previous studies have highlighted a poor dissemination of evidence to support GPs’ decision-making [17]. Indeed, there is scope for improving the use of therapy through such a tool; preliminary data evaluating the CARAT as a screening tool shows that, in a cohort of older patients (≥65 years) with AF, less than half (44%) of those indicated for warfarin (N = 126) were prescribed it; in those in whom warfarin was NOT indicated (i.e., unfavorable risk: benefit profile), 46% of patients *were* actually prescribed it. Furthermore, 12% of patients did not receive any preventative therapy at all, despite it being indicated and the absence of contraindications (30). The National Institute of Clinical Studies acknowledges our earlier findings and those of others, supporting the crucial need for improving stroke risk assessment and provision of information to GPs [30].

### Study aim and objectives

Optimizing stroke prevention in general practice requires targeted interventions to address the critical need for improving stroke risk assessment and provision of information to GPs [30]. Therefore, this study will undertake a trial of the CARAT in general practice as a targeted intervention to assist clinicians in selecting appropriate therapy for stroke prevention in older persons with AF.

The **Specific Objectives** of this study are to:

1. evaluate the impact of CARAT on the prescription of antithrombotic therapy post-application of the tool, including initiation of, and changes to, therapy (quantitative data analysis)

2. measure the longer-term impact of CARAT on clinical outcomes (strokes, bleeds) (quantitative data analysis)

3. gauge clinician feedback regarding CARAT in terms of its perceived utility in practice (qualitative data analysis)

### Hypotheses

Application of the CARAT in general practice will improve the utilization of appropriate antithrombotic (anti-clotting) therapy in older persons with AF. Further, it will improve clinical outcomes in older persons with AF, specifically in terms of reducing the incidence of stroke and bleeding events.

## Methods and design

### Study design and setting

#### Design

This study is a prospective cluster-randomized controlled clinical trial (CRT) utilizing an innovative online practice tool, i.e., the Computerized Antithrombotic Risk Assessment Tool - CARAT (Trial Registration: ACTRN12613000060741). Approval for the conduct of the study has been granted by the Human Research Ethics Committees of the participating institutions: University of Technology Sydney; University of Sydney; and University of Newcastle. Additionally, support from the participating Divisions of General Practice has also been granted: Hunter Urban (i.e., GP Access), Hunter Rural, Central Coast, and Hornsby Ku-ring-gai divisions.

#### Setting

The study will be conducted in Australia, in the state of New South Wales (NSW). Specifically, the study will be undertaken within 4 Divisions of General Practice that accommodate a high proportion of older patients and which represent persons from diverse backgrounds in terms of socioeconomics, health status, and access to health services: City-Metropolitan (Northern Suburbs), Coastal-Region (Central Coast, NSW), Regional-Urban (Hunter Region, Newcastle metropolitan area), and Regional-Rural (Hunter Region, rural areas).

### Participants

**Figure 1:**
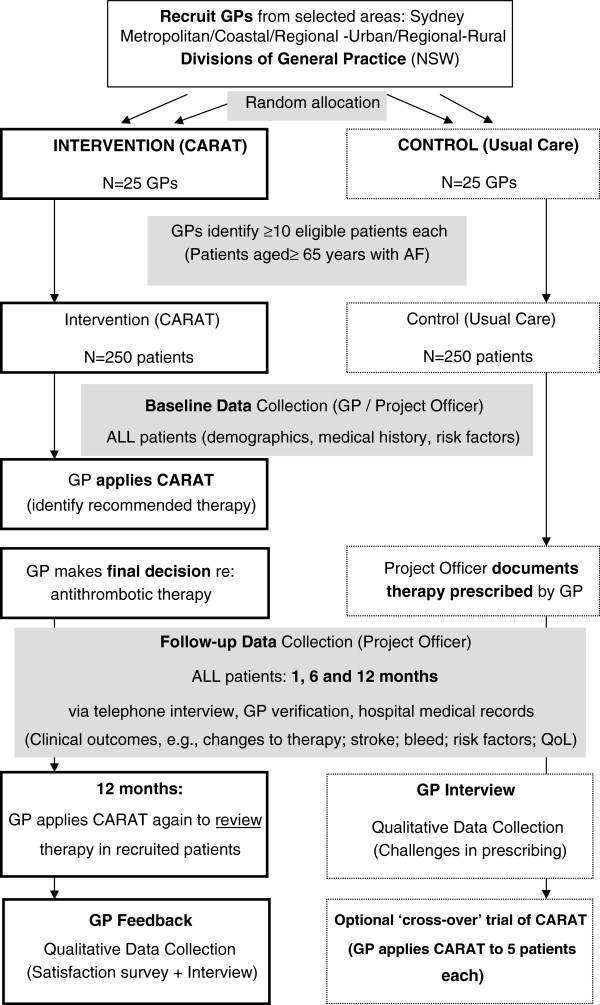
Schematic outline of the main study.

Fifty GPs will be recruited and randomized to either the intervention (CARAT) arm or control (usual care) arm to recruit a total 500 elderly patients with AF (Figure 1), i.e., 25 GPs will be recruited to each of the two study arms. Randomization will occur at the GP level at the time of recruitment [31], not at the division or regional level, to facilitate comparison over a cross-section of GPs and patients, and to negate any regional confounders.

The sample size has been estimated based on previous local data [12,26]. Estimated changes to the utilization of therapy are founded in data from previous studies where only 35% of high-risk AF patients were prescribed warfarin, where interventions have demonstrated upgrades in therapy in poorly protected patients by at least 15% to 23% [12,26]. Taking into account any clustering effects (*a prior* intra-cluster correlation/ICC = 0.03), and a participant drop-out rate of up to 20%, a minimum of 25 GPs and 250 patients are required to achieve statistically significant results [32,33].

### Participant recruitment and eligibility

a) General Practitioner

GPs will be recruited via invitational letters, promotional flyers inside newsletters, and presentations during education events, coordinated by the Divisions of General Practice. Eligible GPs include those who:

•are practicing in one site at a general practice surgery and not across multiple sites or surgeries;

•practice in the specified Divisions of GP;

•provide informed written consent to participate in the study.

Once recruited, the GPs will be randomly allocated to a study arm (‘intervention’ versus ‘control’) using computer-generated random allocation number sequences. The process will be coordinated centrally by a Chief Investigator independently of GP recruitment, intervention implementation, or data collection processes, and who will conceal the initial allocation process from project officers (e.g., allocation numbers will be placed in sealed, serially coded envelopes, and housed in a locked cabinet).

b) Patient

Patients will be recruited by their GPs during routine care over a 3-month period. Each GP will be required to recruit 10 patients that meet the following study inclusion criteria:

•are aged ≥ 65 years;

•have a diagnosis of chronic (persistent) AF, whether new or pre-existing and irrespective of the antithrombotic therapy prescribed at the time of recruitment;

•provide informed written consent to participate in the study and agree to be followed-up via telephone interview over a 12 month period (1,6 and 12 months).

Patients will be informed that all personal information is de-identified and that they may withdraw from the study at any time without any negative consequences.

### Minimizing selection bias and contamination

To avoid selection bias (during the recruitment process) and potential contamination post-randomisation, GPs will be generically informed that 1) the study targets the challenges of prescribing therapy in older patients and 2) what participation entails in terms of time, resources, and remuneration; specific detail about the intervention (hypotheses, CARAT) will be provided to Group A (intervention) after GPs are randomly allocated (concealed allocation) to minimise performance bias.

Although the gold-standard approach to evaluating behavioural interventions [34], it is inherently impossible to achieve complete blinding in such CRTs as the intervention cannot be masked [35,36]. Here, blinding is feasible as follows:

•GPs will be blinded in part (ethically) to their allocation status through the use of alternate terminology (i.e., avoiding the terms “intervention” and “control”, and instead using ‘Group A’ or ‘Group B’), thereby reducing performance bias.

•Researchers (Chief Investigators) will be blinded to the allocation status of subjects during analyses (i.e., blinded assessment, e.g., PROBE design)

•project officers (i.e., the research assistants) will be blinded to the initial allocation process (i.e., concealed allocation of GPs will be independently undertaken by the chief investigator) to minimise pre-randomisation selection bias. However, the project officers will be subsequently unblinded to the different procedures between study arms as they will necessarily liaise with GPs in both arms to collect data and provide support for the intervention (CARAT)

•assessment bias will be minimised by using common objective and standardised data collection instruments in both study arms and avoiding the terms “intervention” and “control” (using Group A or B) during recruitment and data collection

•patients will be blinded to the study allocation (control versus intervention) of their GP; in consenting to data collection and follow-up, patients will be informed of the specific study hypotheses relating to monitoring therapy prescribed and clinical outcomes over time.

In using the cluster-randomized trial design, contamination *within* groups of GPs is minimized. Any contamination *between* groups will be minimized through specific screening during the recruitment process to exclude those GPs practising across multiple (≥1) surgeries. Within ‘group’ practices (e.g., medical centres where more than 1 GP is employed), all participating GPs will be allocated to the same study arm (block randomisation). Once allocated, drop-ins between arms and sharing of the ‘intervention (i.e., password protected CARAT) will not be permitted.

### Intervention Arm

The CARAT will be disseminated via a web-based interface to GPs randomized to the intervention arm. Upon recruitment, GPs will be given access to CARAT via log-ins, passwords, together with supporting information and on-site training by the project officer.

During routine clinical practice, GPs will utilize the CARAT to support their decision-making processes in selecting and/or reviewing antithrombotic therapy in their patients. This will involve performing an individualized risk assessment and generating a treatment recommendation for their patient by carefully assessing relevant risk factors for stroke, bleeding, medication misadventure and entering this data, as guided by CARAT, into the web-based platform. CARAT will then quantify the patient’s estimated risk of stroke versus bleeding, flag pertinent medication management issues, and generate a recommendation for therapy (warfarin, aspirin, none, other). *Note: CARAT does NOT require identifiable personal information to be entered NOR is any information saved to the website.*

The GP will consider the CARAT output and make a final treatment decision to initiate, cease, or maintain current antithrombotic therapy. Where the GP may disagree with CARAT, the rationale for the decision will be documented in the data collection process. Reviewed patients will be followed-up for up to 12 months to document key clinical outcomes (i.e., strokes, bleeds).

### Control Arm

GPs randomized into the control arm will follow their usual care practices. They will review and select antithrombotic therapy using their own clinical judgment, processes and resources. At completion of the study (i.e., final patient follow-up period at 12 months is complete), the GPs in the control arm will be given the opportunity to trial the CARAT on 5 of their patients in their practice setting (‘cross-over’ arm) to canvas feedback on the tool.

### Data collection

In addition to the output generated by CARAT, purpose-designed data collection instruments (record forms, surveys, questionnaires) will be used to extract and record specific data (Table 1). Dedicated Project Officers will be allocated to each participating division of general practice, to coordinate the conduct of the study (i.e., participant recruitment, implementation of intervention, data collection, provision of support to GPs) at each site.

**Table 1:** Data collection items for GPs and patients

**GPs**	**Patients**
• Demographics (age, gender)	• Patient demographics
• Practice experience (years, specialty)	• Medical history, co-morbidities, medication
• Practice site features (location, number of clients)	• History of AF (duration, new onset or chronic)
• Decision-making processes (e.g., resources or processes normally used)	• Risk factors for stroke (per CARAT)
• Agreement with CARAT recommendations (Intervention group)	• Risk factors bleeding (per CARAT)
• Satisfaction with CARAT as a tool (Intervention group)	• Quality of life (e.g., SF36)
• Barriers to prescribing therapy (ALL GPs)	• Risk factors for medication misadventure [25]
	• Current antithrombotic therapy utilised
	• History of clotting and bleeding events
	• Health services used to support therapy
	• Health services used to support therapy

Baseline data for patients and GPs will be collected by the project officer within 48 hours of the index consultation. During the baseline visit, the GP will apply CARAT to consecutive admissions over a period of 3 months and obtain the patient’s consent to participate. GPs will print-out the completed risk-assessment template (generated by the CARAT) for subsequent review by the project officer. Each GP will then ‘flag’ the patient for review and baseline data collection by the project officer by returning a purpose-designed fax-back form with basic patient details. Baseline data will be extracted from medical notes, patient interview, and a brief patient survey.

Clinical outcome data will be collected at 1, 6 and up to 12 months after the index consultation. This data will be collected from patients via follow-up telephone interview (15–20 minutes) by the project officer. To verify outcomes, the GP will be contacted via phone and/or the patient’s notes reviewed. Hospital medical records may also be reviewed where patients have been admitted for an acute event including a stroke or bleed.

A 1-month pilot study will be conducted prior to the main trial to confirm project procedures/logistics.

### Data analysis

Computerized data analysis will employ SPSS (Statistical Package for the Social Sciences) Version 15. ANOVA will test for differences in continuous variables. The Chi-square test will examine differences in independent proportions. Kappa analysis will detect the level of agreement between prescribers’ clinical judgment and the CARAT output. Logistic regression analysis will identify predictors of clinical events (clotting versus bleeding), and selection of antithrombotic therapy, accounting for the use of the CARAT. Survival analysis will assess mortality rates in both arms. All analyses will necessarily be based on the ITT principle; standard imputation methods (e.g., last observation carried forward) will be used to estimate missing data (withdrawal, failure to start) and data analysed according to initial group (random) allocation. All analyses will be adjusted for the effects of clustering, and set at a significance (p) level of 0.05.

### Outcomes measured

The primary outcome measures for the intervention will include:

•changes to therapy (initiations, changes, cessations) made following application of CARAT;

•difference in the proportion of patients receiving no therapy;

•overall difference in the proportion of patients receiving antithrombotic therapy;

•difference in the proportion of patients receiving warfarin therapy;

•difference in the proportion of patients experiencing adverse clinical outcomes (ALL events).

The secondary outcome measures will include:

•the sustainability of prescribed therapy over time, i.e., changes to antithrombotic therapy made during patient follow-up up to 12 months;

•incidence of any clotting (thromboembolism, stroke) events up to 12 months;

•incidence of any bleeding (minor or major hemorrhage) events up to 12 months.

### Qualitative data collection and analysis

Qualitative data is integral to this study; it will triangulate the findings regarding the utility and refinement of CARAT for future sustainable implementation in general practice (previous data focused on CARAT’s utility in the acute care/hospital setting).

In-depth feedback will be obtained via individual face-to-face or telephone interviews. A semi-structured process will be adopted using a purpose-designed interview guide. Initially, 5 to 10 GPs in each arm will be interviewed until theme saturation is achieved. Each face-to-face interview will be digitally-recorded, transcribed verbatim, and then thematically analysed using inductive coding [37].

## Discussion

The need for tailored sustainable interventions to optimize the use of antithrombotic therapy for stroke prevention in at-risk patients is well documented [26], which will ultimately reduce the considerable burden of stroke suffered by individuals and the global community, on the background of an ageing population. In this study, a novel decision-support tool will be tested, to assist clinicians (specifically, GPs) in their decision-making.

Given the expansion of the treatment armamentarium following the development of the NOACs, the decision-making will necessarily become more complex, and will additionally be driven by the economic implications of the potentially widespread use of newer, more expensive therapies. This study specifically considers those therapies that have been historically available in the local practice arena, for which there is vast and robust evidence-base (i.e., warfarin, aspirin). However, the findings of this study will inform the need to integrate newer therapies, and their specific benefits and limitation, within decision-support tools. Separate to evaluating the impact of this tool as a targeted intervention, this study will additionally provide data on the absolute and relative risks of clinical outcomes (e.g., changes to therapy; stroke; bleeds; mortality) in an Australian cohort of older persons with AF, and identify predictors of these (e.g., risk factors, antithrombotics prescribed).

Our unique approach in this study addresses National Research Priorities in Preventative Health and targets major health issues in effective health care including an ageing population, burden of chronic illness, and translation of evidence into practice. A key feature of this study is that it targets the interface of patient care (general practice), addresses the at-risk population, and provides evidence for the impact of the intervention by evaluating related clinical outcomes. Its innovation lies in the application of a novel computerized antithrombotic risk assessment tool, developed by the researchers, to provide GPs not only with a screening instrument, but importantly a mechanism for comprehensive risk assessment that, for the first time, quantifies the risk versus benefit of antithrombotic therapy in individual patients. Eventually, the CARAT may be integrated into prescribing software, and/or other primary care services including Medication Review and Disease State Management rendering its application in practice highly sustainable.

## Abbreviations

AF: Atrial fibrillation; ANOVA: Analysis of variance; CARAT: Computerized antithrombotic risk assessment tool; GP: General practitioner; SF36: 36-Item Short-Form Health Survey; SPSS: Statistical package for the social sciences.

## Competing interests

The authors declare that they have no competing interests.

## Authors’ contributions

BB conceived the project, coordinated the design of the study, and prepared the manuscript. PM participated in the study design, particularly in relation to aspects of implementation and patient care in general practice, as well as drafting of the manuscript. SH participated in the study design, focusing on aspects relating to the management of older persons and decision-making, and helped draft the manuscript. IK participated in the study design, informing the methodological approaches and data analyses to be used, as well as preparation of the manuscript. All authors have read and approved the final manuscript.

## Authors’ information

The authors comprise a multidisciplinary team of health-trained academics, with specific expertise in implementing intervention to improve the utilization of evidence-based therapies in at-risk patients through targeted interventions. BB is an Academic Pharmacist from the Graduate School of Health- School of Pharmacy, University of Technology, Sydney and Royal North Shore Hospital. PM is an Academic GP from the Discipline of General Practice, University of Newcastle. IK is an Academic Researcher from the Faculty of Pharmacy, University of Sydney. SH is a Clinical Pharmacologist and Geriatrician from the Faculty of Medicine, University of Sydney, and Royal North Shore Hospital.

## Pre-publication history

The pre-publication history for this paper can be accessed here:

http://www.biomedcentral.com/1472-6963/14/55/prepub
